# Women and trachoma: why prioritising gender equity is essential to achieve vision for all

**Published:** 2023-07-07

**Authors:** Angelia Sanders, Paul Emerson, Paul Courtright, PJ Hooper

**Affiliations:** 1 Associate Director, Trachoma Control Program, The Carter Center, and Immediate Past Chair, International Coalition for Trachoma Control, Atlanta, USA.; 2 Director, International Trachoma Initiative, Atlanta, USA.; 3 Board Chair, Kilimanjaro Centre for Community Ophthalmology, San Diego, USA.; 4 Deputy Director, International Trachoma Initiative, and Chair, International Coalition for Trachoma Control, Atlanta, USA.

**The second edition of the Women and Trachoma manual provides updated strategies for gender-sensitive and equitable national trachoma programmes.**

**Figure:**
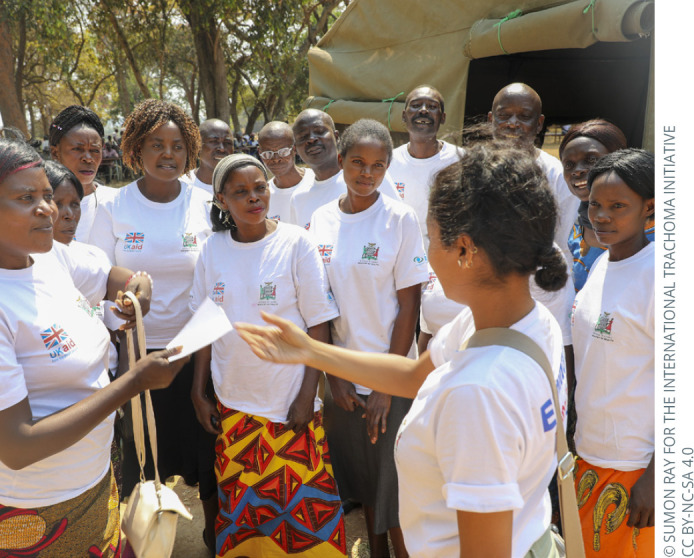
Gender representation in the eye care workforce can make a positive difference. **ZAMBIA**

Women are almost twice as likely as men to require surgery to treat trachomatous trichiasis (TT), the blinding stage of trachoma, and the leading infectious cause of blindness worldwide. This is, in part, caused by women’s increased exposure to *Chlamydia trachomatis*, the bacterium that causes trachoma, due to gender norms which typically place them in the role of caregiver within the home. The effects of repeated trachoma infections are often compounded by barriers to quality healthcare and lower uptake of services, which increase health inequalities and threaten progress towards the elimination of trachoma as a public health problem and the achievement of universal eye health coverage.

In April 2023, the second edition of the Women and Trachoma manual^1^ was launched. The manual provides updated knowledge, skills, strategies and lessons learned from trachoma-endemic countries across multiple WHO regions to enable the development of gender-sensitive national trachoma programmes and ensure equitable access to all aspects of the World Health Organization (WHO)-endorsed SAFE strategy for trachoma elimination (surgery, antibiotics, facial cleanliness, and environmental improvement).

A key lesson from the manual is the importance of gender representation and diversity in the health care workforce, which is often complicated by women having fewer educational and work opportunities in many trachoma-endemic settings. However, women often know, first-hand, the challenges faced by other women and girls in their communities and how to best support them with trachoma treatment and prevention. Examples from Nigeria show that, in some communities, traditional or religious customs mean male case finders (community members trained to detect potential cases of TT) cannot enter a household unless there is a man at home. In these settings, women are at risk of missing out on surgery simply because the case finder is male.

The manual recommends inclusivity throughout all levels of the recruitment process to support the participation of women in the trachoma workforce. Recruitment through local authorities (regional or district-level ministries of health) and community leaders improves the level of local ownership and participation. In turn, this can ensure stronger female representation in paid roles such as team supervisors, drug distributors, TT case finders, and community mobilisers.

The manual recognises that progress has been made to advance gender equity by recording people’s gender at the point of service. Collecting such gender-disaggregated data has enabled national programmes to monitor their effectiveness in reaching each group, allowing them to be more targeted in their approach. Programmes could also further disaggregate their data to identify gender gaps specific to a particular subgroup, including people with disabilities, religious or ethnic minorities, or nomadic populations. In doing so, national programmes will be able to consider the intersections of gender and other social, cultural, and socioeconomic attributes that affect access to care.

The lessons provided throughout the manual are essential to achieve the target set in the global NTD road map,^2^ published by WHO: the elimination of trachoma as a public health problem by 2030. In addition, the manual emphasises the importance of gender sensitivity as trachoma interventions are integrated into routine systems, which will be necessary in a post-elimination setting. Routine clinical eye care services may already experience gender inequality and, as a result, more is needed to support routine TT services. This manual will help eye care programme personnel to assess all aspects of their service delivery model and identify strategies to address gender inequity.

The *Lancet Global Health* Commission on Global Eye Health,^3^ published in 2021, emphasised that improving eye health contributes to promoting gender equity (Sustainable Development Goal 5) and reduced inequalities. The Women and Trachoma manual provides actionable guidance and lessons that, while written for trachoma stakeholders, remain relevant for many other eye health conditions. To achieve vision for all, including the elimination of trachoma as a public health problem, we must work within community structures to elevate, profile, and include women in everything we do. We must deliberately target women and girls to ensure they have the necessary tools and access to resources to prevent and treat trachoma in themselves, their families, and their communities.
